# Dietary index for gut microbiota and odds of gestational diabetes mellitus: associations with inflammatory and metabolic biomarkers

**DOI:** 10.3389/fnut.2026.1895387

**Published:** 2026-08-10

**Authors:** Yi Su, Xiuping Du, Zhiying Song, Xifeng He

**Affiliations:** Department of Obstetrics and Gynecology, Shanxi Children’s Hospital (Shanxi Maternal and Child Health Hospital), Taiyuan, Shanxi, China

**Keywords:** diet, gestational diabetes mellitus, gut microbiome, inflammation, insulin resistance

## Abstract

**Background:**

Gestational diabetes mellitus (GDM) is a common metabolic disorder of pregnancy associated with adverse maternal and neonatal outcomes. Emerging evidence suggests that dietary patterns supportive of gut microbial health and associated systemic inflammation may contribute to the pathophysiology of GDM. This study evaluated the association between a dietary index oriented toward gut microbiota (DI-GM)—comprising foods previously linked to beneficial gut microbial composition—and GDM odds, metabolic profiles, inflammatory markers, and pregnancy outcomes.

**Methods:**

This case–control study included 800 pregnant women (400 GDM cases and 400 controls) at 24–28 weeks of gestation. Dietary intake was assessed using a validated food frequency questionnaire, and DI-GM scores were derived based on intake of microbiota-supportive and disruptive foods. Biochemical measurements included glucose parameters, lipid profile, hs-CRP, IL-6, TNF-*α*, and lipopolysaccharide. Multivariable logistic regression and structural equation modeling were used for association and mediation analyses.

**Results:**

Women with GDM had lower DI-GM scores than controls (7.09 ± 1.64 vs. 9.52 ± 1.83, *p* < 0.001). In the fully adjusted model (Model 2), women in the highest DI-GM tertile (T3) had 55.4% lower odds of GDM compared with those in the lowest tertile (T1) (OR = 0.446). A significant inverse trend was observed across DI-GM tertiles (*P* trend = 0.002), indicating a dose–response relationship. Each 1-unit increase in DI-GM score was associated with an 11.0% lower adjusted odds of GDM (OR = 0.890); however, the association appeared to be nonlinear, with the strongest protective effect observed in the highest tertile. These findings suggest a potential threshold effect, whereby clinically meaningful benefits become evident primarily at higher levels of dietary adherence. The highest DI-GM tertile demonstrated lower fasting glucose, HbA1c, HOMA-IR, triglycerides, hs-CRP, and LPS, alongside higher HDL-C (all *p* < 0.05). Among women with GDM, higher DI-GM scores were strongly associated with lower rates of cesarean delivery, preeclampsia, gestational hypertension, macrosomia, neonatal hypoglycemia, NICU admission, and respiratory distress syndrome (all *P*-trend < 0.001). However, in the control group, this protective trend was not observed for any outcome except for a paradoxical significant increase in cesarean delivery with higher DI-GM adherence (*P*-trend < 0.001), while all other outcomes showed no significant trends (*p* > 0.10). Exploratory mediation analyses suggested hs-CRP may partially explain the DI-GM-glycemic association, but concurrent measurement precludes causal mediation. The negative indirect effects indicate an inconsistent mediation pattern, suggesting hs-CRP may not function as a classical mediator but rather reflects shared metabolic pathways or residual confounding. These findings are exploratory and hypothesis-generating, not confirmatory.

**Conclusion:**

Greater adherence to a microbiota-oriented dietary pattern was associated with lower GDM odds and improved maternal–neonatal outcomes. However, without direct gut microbiota measurement, these findings represent associations—not confirmation—of diet–microbiota–inflammation pathways, and require further investigation.

## Introduction

Gestational diabetes mellitus (GDM) is one of the most common metabolic complications during pregnancy and represents a growing global public health concern (1). The prevalence of GDM has increased substantially over recent decades, largely due to rising maternal obesity, sedentary lifestyle, advanced maternal age, and unfavorable dietary habits (2). GDM is characterized by glucose intolerance with onset or first recognition during pregnancy and is associated with multiple adverse maternal and neonatal outcomes, including preeclampsia, cesarean delivery, excessive gestational weight gain, macrosomia, neonatal hypoglycemia, respiratory distress syndrome, and increased risk of future type 2 diabetes mellitus in both mother and offspring (3). In addition, accumulating evidence suggests that metabolic disturbances during pregnancy may exert long-term programming effects on offspring metabolic health and cardiometabolic risk (4).

Although the precise pathophysiology of GDM remains incompletely understood, insulin resistance, chronic low-grade inflammation, oxidative stress, and altered metabolic homeostasis are considered major contributing mechanisms (5). Physiological insulin resistance normally increases during pregnancy to support fetal growth; however, exaggerated insulin resistance combined with impaired pancreatic β-cell compensation may result in hyperglycemia and GDM development (6). Several inflammatory mediators, including high-sensitivity C-reactive protein (hs-CRP), interleukin-6 (IL-6), and tumor necrosis factor-alpha (TNF-*α*), have been implicated in the progression of insulin resistance and glucose dysregulation during pregnancy (7). Moreover, obesity-related metabolic abnormalities and adipokine imbalance may further aggravate inflammatory responses and impair insulin signaling pathways (8).

Recently, increasing attention has focused on the potential role of gut microbiota in the development of metabolic diseases, including obesity, insulin resistance, and GDM (9, 10). The maternal gut microbiota undergoes substantial physiological alterations during pregnancy, and dysbiosis may contribute to impaired glucose metabolism through multiple mechanisms, including increased intestinal permeability, endotoxemia, systemic inflammation, and modulation of host energy metabolism (11). Biomarkers such as lipopolysaccharide (LPS), and zonulin have emerged as potential indicators of gut barrier dysfunction and microbiota-related metabolic alterations (12). Several studies have suggested that women with GDM exhibit reduced microbial diversity and altered abundance of beneficial bacterial species compared with normoglycemic pregnant women (13).

Diet is a key determinant of gut microbiota composition and metabolic function (14). Diets rich in fruits, vegetables, legumes, whole grains, nuts, fermented foods, olive oil, and fiber promote microbial diversity, and anti-inflammatory pathways (15) whereas refined carbohydrates, processed meat, ultra-processed foods, saturated fat, and sugar-sweetened beverages promote dysbiosis, endotoxemia, and systemic inflammation (16). Despite growing recognition of the gut microbiota–metabolism axis, most previous studies have focused on individual nutrients or conventional dietary indices rather than comprehensive microbiota-oriented patterns (17–19), and evidence linking diet, gut-derived metabolites, inflammatory biomarkers, and maternal-neonatal outcomes in GDM remains limited. Few studies have simultaneously examined microbiota-supportive and microbiota-disruptive dietary components with metabolic, inflammatory, and gut permeability markers, and the mediating role of systemic inflammation in microbiota-oriented dietary adherence and glycemic regulation during pregnancy has not been sufficiently explored (20).

Therefore, the present study aimed to investigate the association between adherence to a Gut Microbiota-Oriented Dietary Index (DI-GM) and the risk of gestational diabetes mellitus in pregnant women (21). In addition, we evaluated the relationships between DI-GM adherence and glycemic parameters, insulin resistance indices, inflammatory biomarkers, gut-derived metabolites, lipid profile, maternal metabolic characteristics, and neonatal outcomes (22, 23). We further examined the potential mediating role of systemic inflammation, particularly hs-CRP, in the association between DI-GM and glycemic outcomes. To our knowledge, this is among the first studies to comprehensively evaluate a microbiota-oriented dietary index in relation to inflammatory, metabolic, and neonatal parameters simultaneously in women with GDM. Importantly, we also assessed whether DI-GM adherence is associated with clinically relevant obstetric complications that are mechanistically linked to hyperglycemia, including preeclampsia, gestational hypertension, and adverse neonatal outcomes.

## Methods

### Study design and participants

This case–control study was conducted among pregnant women attending the prenatal endocrinology, obstetric, and maternal-fetal medicine clinics of Shanxi Children’s Hospital and affiliated maternal healthcare centers in Taiyuan, China, between May 2025 and April 2026. A total of 800 pregnant women were enrolled, including 400 women with gestational diabetes mellitus (GDM) and 400 normoglycemic pregnant controls. Cases and controls were frequency-matched according to maternal age and gestational age to minimize potential confounding effects. This study followed the STROBE guidelines for reporting observational studies.

Eligible participants were women aged 18–45 years with singleton pregnancies who were enrolled at 10–14 weeks of gestation. GDM screening was performed at 24–28 weeks of gestation. Participants were not aware of their glycemic status at the time of dietary assessment, as GDM screening had not yet been performed. GDM was diagnosed using a standardized 75-g oral glucose tolerance test (OGTT) according to the International Association of Diabetes and Pregnancy Study Groups (IADPSG) criteria. Women were classified as having GDM if one or more of the following thresholds were met: fasting plasma glucose ≥92 mg/dL, 1-h plasma glucose ≥180 mg/dL, or 2-h plasma glucose ≥153 mg/dL (24).

Controls consisted of normoglycemic pregnant women attending the same clinical centers during the same recruitment period who did not meet GDM diagnostic criteria. Controls were selected from the same source population as cases to reduce selection bias.

**Figure 1:**
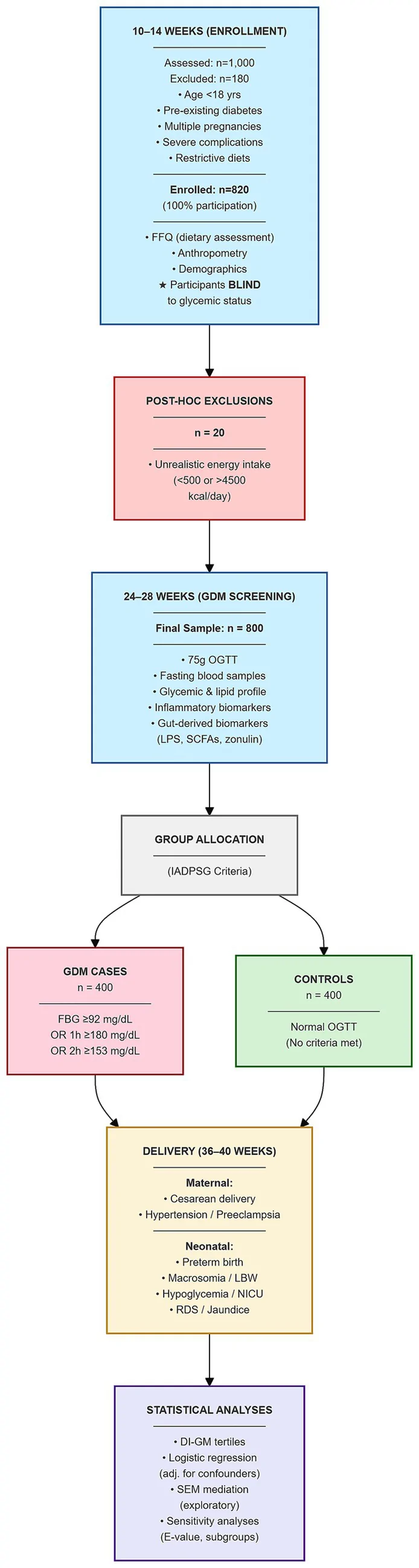
Presents the combined schematic timeline and participant flow diagram, illustrating the temporal sequence of enrollment (10–14 weeks), dietary assessment (FFQ), GDM screening (24–28 weeks), and delivery outcomes (36–40 weeks).

Exclusion criteria included pre-existing type 1 or type 2 diabetes mellitus, chronic renal disease, hepatic disorders, autoimmune diseases, malignancy, inflammatory bowel disease, multiple pregnancy, severe pregnancy complications diagnosed before recruitment, adherence to medically prescribed restrictive diets, and incomplete dietary or biochemical data. Women with implausible total energy intake values (<500 or >4,500 kcal/day) were additionally excluded from the analysis. Participants were recruited using a consecutive sampling strategy during routine prenatal visits at 24–28 weeks of gestation. Among 1,000 women initially screened, 180 were excluded before enrollment (age <18 years, pre-existing diabetes, multiple pregnancies, severe pregnancy complications diagnosed before recruitment, or medically prescribed restrictive diets), leaving 820 eligible women. All 820 eligible women agreed to participate. After data collection, 20 participants were excluded because of implausible total energy intake values (<500 or >4,500 kcal/day). The final analyzed sample comprised 800 women (400 GDM cases and 400 controls). Figure 1 presents the combined schematic timeline and participant flow diagram, illustrating the temporal sequence of enrollment (10–14 weeks), Food Frequency Questionnaire (FFQ), GDM screening (24–28 weeks), and delivery outcomes (36–40 weeks).

The study protocol was approved by the Medical Research Ethics Committee of Shanxi Children’s Hospital (Shanxi Maternal and Child Health Hospital) (Approval No. IRB-WZ-2025-045). Written informed consent was obtained from all participants prior to enrollment. The study was conducted in accordance with the Declaration of Helsinki and relevant ethical standards for human research.

### Sample size

Sample size was estimated based on previous observational studies evaluating dietary patterns and GDM risk. Assuming a GDM prevalence of 15% in the source population, an OR of 0.60, 80% power, *α* = 0.05, and a 1:1 case–control ratio, the required sample size was 380 per group (760 total). After adding 5% for missing data, we targeted 800 participants (25).

### Data collection and general assessments

Demographic, socioeconomic, lifestyle, and obstetric information were collected using structured interviewer-administered questionnaires completed during 24–28 weeks of gestation by trained nutritionists and healthcare personnel. Collected variables included age, educational level, occupation, household income, socioeconomic status, residency, marital status, smoking, alcohol consumption, parity, gravidity, abortion history, previous history of GDM, family history of diabetes, hypertension, polycystic ovary syndrome (PCOS), thyroid disorders, anemia, medication use, antibiotic use, probiotic supplementation, sleep duration, and physical activity. Information on prenatal antibiotic exposure and probiotic supplementation was collected via structured questionnaire; responses were recorded as binary variables (yes/no) indicating any use during the current pregnancy. Details on specific antibiotic classes, dosage, frequency, or duration of use were not collected.

Physical activity was assessed using the validated International Physical Activity Questionnaire (IPAQ) short form, which has been validated for use in pregnant Chinese populations. Total physical activity was expressed as metabolic equivalent task (MET) hours/week, calculated by multiplying the MET coefficient for each activity type (walking: 3.3 METs; moderate intensity activity: 4.0 METs; vigorous intensity activity: 8.0 METs) by the duration in hours per week and summing across activities (26). The questionnaires were administered by trained interviewers at enrollment (10–14 weeks of gestation).

To minimize interviewer and information bias, all questionnaires were administered by trained personnel using standardized operating procedures. Laboratory analysts and outcome assessors were blinded to dietary exposure categories and GDM status during biomarker quantification and outcome assessment. Demographic, socioeconomic, lifestyle, and obstetric information were collected using structured interviewer administered questionnaires completed at enrollment (10–14 weeks of gestation) by trained nutritionists and healthcare personnel.

### Anthropometric and clinical measurements

Anthropometric assessments were performed at two standardized time points: enrollment (10–14 weeks of gestation) and the GDM screening visit (24–28 weeks of gestation). At both visits, body weight was measured to the nearest 0.1 kg using calibrated digital scales with participants wearing light clothing and no shoes. Height was measured to the nearest 0.1 cm using a wall mounted stadiometer at enrollment. Pre-pregnancy BMI was calculated using self-reported pre-pregnancy weight and measured height (kg/m^2^). Gestational weight gain (GWG) was calculated as the difference between pre-pregnancy weight and body weight measured at 24–28 weeks.

Blood pressure measurements were obtained after at least 10 min of seated rest using a calibrated sphygmomanometer. Two measurements were recorded at 5-min intervals, and the mean systolic blood pressure (SBP) and diastolic blood pressure (DBP) values were used in the final analyses. Pulse rate was additionally recorded during clinical assessment.

### Dietary assessment

Dietary intake was assessed prospectively at enrollment (10–14 weeks of gestation) using a validated semiquantitative food frequency questionnaire (FFQ) specifically adapted for pregnant women. The FFQ assessed habitual dietary intake over the preceding 3 months (approximately 7–14 weeks of gestation) to capture dietary patterns before GDM screening (27). Participants completed the FFQ before the oral glucose tolerance test and were unaware of their glycemic status at the time of dietary assessment. The FFQ included 82 commonly consumed food items and was administered by trained nutritionists using standardized protocols. Participants reported the frequency of food consumption on daily, weekly, or monthly bases, and portion sizes were converted into grams using standard household measures. The FFQ has been validated for use in Chinese pregnant populations, with reproducibility correlation coefficients ranging from 0.53 to 0.82 and validity coefficients of 0.45–0.67 across nutrient comparisons (26). Daily nutrient intakes including total energy, macronutrients, dietary fiber, sugar, fructose, cholesterol, fatty acids, and micronutrients were calculated using nutrition analysis software (Nutrition Star, version 2.0) based on the Chinese Food Composition Database. Food groups including fruits, vegetables, legumes, whole grains, refined grains, fermented foods, yogurt, kefir, pickles, nuts, seeds, olive oil, fish, poultry, red meat, processed meat, fast food, ultra-processed foods, sugar sweetened beverages, tea, coffee, and water intake were additionally quantified. Participants with implausible total energy intake values (<500 or >4,500 kcal/day) were excluded from the analysis.

### Calculation of gut microbiota-oriented dietary index (DI-GM)

The Gut Microbiota-Oriented Dietary Index (DI-GM) was adapted from Kase et al. (21), to align with the food composition database and dietary patterns of the Chinese pregnant population. The index comprises 14 food items selected based on published evidence for their association with gut microbiota composition and metabolic regulation, according to four criteria: (1) prebiotic or postbiotic effects (e.g., dietary fiber, fermented dairy, soybeans, whole grains); (2) high polyphenol content (e.g., green tea, coffee, cranberries, broccoli, avocado); (3) favorable effects on gut barrier function (e.g., chickpeas, legumes); and (4) established associations with reduced inflammation and improved metabolic health. Beneficial components included broccoli, avocado, coffee, chickpeas, cranberries, fermented dairy, dietary fiber, green tea, soybeans, and whole grains. Adverse components comprised high-fat diet (≥40% energy from fat), red meat, processed meat, and refined grains. These components were selected as they were specifically represented in the FFQ and previously identified as distinctive components of the microbiota-oriented dietary pattern. For beneficial components, a score of 1 was assigned if intake was at or above the median, and 0 otherwise; for adverse components, scoring was reversed. This median-based approach is consistent with the original DI-GM (21) and avoids prespecified cut points that may not be generalizable (Supplementary Table S4). All median values were calculated using the entire study population (n = 800) before outcome analyses, independent of case–control status, thus avoiding differential score construction between cases and controls. Individual component scores were summed to generate the overall DI-GM score (range 0–14), and participants were categorized into tertiles (T1–T3). Higher scores reflected greater adherence to a gut microbiota-supportive dietary pattern. Although this version has not been previously validated in pregnant women, the FFQ has been validated in Chinese pregnant populations (27), and the components are consistent with the original index (22, 23). Internal consistency was not assessed, as the index was constructed as a cumulative score of empirically supported components rather than a latent construct.

### Biochemical assessments

After overnight fasting for 10–12 h, venous blood samples were collected during 24–28 weeks of gestation concurrently with routine GDM screening procedures. Blood samples were centrifuged immediately, and serum/plasma aliquots were stored at −80 °C until laboratory analysis. Laboratory analysts were blinded to participant GDM status and dietary group assignment. Dietary interviewers were not blinded to GDM status (as GDM was diagnosed after dietary assessment), but outcomes assessors were blinded to all exposure data.

Fasting blood glucose (FBG), triglycerides (TG), total cholesterol, high-density lipoprotein cholesterol (HDL-C), low-density lipoprotein cholesterol (LDL-C), were measured using automated enzymatic colorimetric assays with commercially available laboratory kits on a fully automated biochemistry analyzer. Commercial enzyme-linked immunosorbent assay (ELISA) kits were obtained from Bioassay Technology Laboratory (Shanghai, China) and Elabscience Biotechnology Inc. (Wuhan, China). Automated biochemical assays were performed using Roche Diagnostics reagents (Basel, Switzerland).

Fasting insulin and C-peptide concentrations were measured using enzyme-linked immunosorbent assay (ELISA) kits (Mercodia AB, Uppsala, Sweden). Serum interleukin-6 (IL-6) and tumor necrosis factor-*α* (TNF-α) were quantified using Quantikine^®^ high-sensitivity ELISA kits (R&D Systems, Minneapolis, MN, United States). High-sensitivity C-reactive protein (hsCRP) was measured using a high-sensitivity ELISA kit (DRG International Inc., Springfield, NJ, United States). Serum zonulin concentrations were determined using a competitive ELISA kit (Immundiagnostik AG, Bensheim, Germany). Circulating lipopolysaccharide (LPS) levels were quantified using a chromogenic Limulus Amebocyte Lysate (LAL) assay (Lonza, Walkersville, MD, United States). Intra-assay coefficients of variation ranged from 4.2 to 7.8% across all biomarkers, while inter-assay coefficients of variation ranged from 7.9 to 11.8%, with the highest variability observed for LPS (intra-assay: 7.2%, inter-assay: 11.8%) and the lowest for insulin (intra-assay: 4.2%, inter-assay: 7.9%). All biomarkers demonstrated acceptable assay performance with coefficients of variation maintained within the predefined acceptable limits (<10% for intra-assay and <12% for inter-assay).

A standardized 75-g oral glucose tolerance test (OGTT) was performed following overnight fasting. Plasma glucose concentrations were measured at fasting, 1 h, and 2 h after glucose ingestion. Insulin resistance was estimated using the Homeostatic Model Assessment for Insulin Resistance (HOMA-IR), calculated as:

HOMA-IR = [fasting insulin (μIU/mL) × fasting glucose (mg/dL)]/405.

### Maternal and neonatal outcomes

Maternal and neonatal outcomes were obtained prospectively from hospital medical records and obstetric follow-up documentation after delivery by trained obstetric research personnel blinded to dietary exposure categories.

Maternal outcomes included mode of delivery (vaginal delivery or cesarean section), gestational hypertension, and preeclampsia. Gestational hypertension was defined as systolic blood pressure ≥140 mmHg and/or diastolic blood pressure ≥90 mmHg occurring after 20 weeks of gestation without proteinuria, whereas preeclampsia was diagnosed according to the American College of Obstetricians and Gynecologists (ACOG) criteria.

Neonatal outcomes included gestational age at delivery, infant sex, APGAR scores at 1 and 5 min, macrosomia, low birth weight (LBW), neonatal hypoglycemia, respiratory distress syndrome, and neonatal intensive care unit (NICU) admission. Birth weight was measured immediately after delivery using calibrated neonatal scales and standardized infant meters. Macrosomia was defined as birth weight ≥4,000 g, whereas LBW was defined as birth weight <2,500 g. Neonatal hypoglycemia was diagnosed according to neonatal glucose screening protocols using capillary or venous blood glucose measurements during the first hours after birth. Respiratory distress syndrome and neonatal jaundice were diagnosed by attending neonatologists according to standard clinical criteria. APGAR scores were routinely assessed at 1 and 5 min after delivery by trained delivery room personnel.

### Statistical analysis

Statistical analyses were performed using Stata version 14 (StataCorp, College Station, TX, United States). Missing data were minimal (<5%) and consistent with missing completely at random (MCAR); complete-case analysis was performed without imputation. Outliers were excluded based on ±3 standard deviations for dietary variables. Continuous variables were assessed for normality using histograms and the Kolmogorov–Smirnov test, and are presented as mean ± standard deviation (SD); categorical variables as frequency and percentage. Between-group comparisons used independent *t*-tests and chi-square tests. Participants were categorized into DI-GM tertiles based on the entire study population distribution. Trend analyses across tertiles used one-way ANOVA for continuous variables and chi-square tests for categorical variables. Multivariable logistic regression estimated odds ratios (ORs) and 95% confidence intervals (CIs) for GDM, adjusted for confounders selected based on prior literature. *P* for trend was calculated using median values of tertiles as continuous variables. Multicollinearity was assessed using variance inflation factor (VIF) analysis; all VIF values were <2.5, indicating no substantial multicollinearity.

Mediation analysis using structural equation modeling (SEM) evaluated indirect effects of hs-CRP on the DI-GM-glycemic association (HOMA-IR, fasting glucose, HbA1c), with direct, indirect, and total effects estimated via maximum likelihood. Correction for multiple testing was not applied as analyses were hypothesis-driven; findings should be interpreted with appropriate caution. A two-sided *p* < 0.05 was considered significant. All mediation analyses are exploratory; hs-CRP and glycemic outcomes were measured concurrently at 24–28 weeks, precluding temporal precedence and causal mediation inference. Sensitivity analyses included excluding women with previous GDM, restricting to non-obese participants (BMI < 30 kg/m^2^), and additional adjustment for energy intake. Additional stratified sensitivity analyses were conducted according to prenatal antibiotic exposure (yes/no) and probiotic supplementation (yes/no) to examine whether these microbiotas related factors modified the association between DI-GM adherence and GDM.

## Results

Table 1 shows the baseline characteristics of the study population according to GDM status. Women with GDM had significantly higher pre-pregnancy BMI (29.5 ± 3.8 vs. 27.0 ± 3.3 kg/m^2^, *p* < 0.001), gestational weight gain (15.8 ± 4.4 vs. 12.8 ± 3.6 kg, *p* < 0.001), HbA1c (5.77 ± 0.8 vs. 5.08 ± 0.4%, *p* < 0.001), fasting blood glucose (102.18 ± 16.07 vs. 85.64 ± 11.32 mg/dL, *p* < 0.001), systolic blood pressure (117.1 ± 11.5 vs. 108.3 ± 11.3 mmHg, *p* < 0.001), and diastolic blood pressure (76.5 ± 7.6 vs. 71.0 ± 6.8 mmHg, *p* < 0.001) compared with controls. Family history of diabetes (47.8% vs. 21.5%, *p* < 0.001) and previous GDM (27.3% vs. 9.0%, *p* < 0.001) were also more prevalent among cases. However, age, physical activity, sleep duration, PCOS, and smoking status did not differ significantly between groups. Moreover, women with GDM had significantly lower DI-GM scores than controls (7.09 ± 1.64 vs. 9.52 ± 1.83, *p* < 0.001) (Table 1).

**Table 1:** General characteristics of study participants according to GDM status.

Variable	GDM cases (*n* = 400)	Controls (*n* = 400)	*p*-value
Demographic and Anthropometric			
Age (years)	30.9 ± 4.4	30.6 ± 4.7	0.381
Pre-pregnancy BMI (kg/m^2^)	29.5 ± 3.8	27.0 ± 3.3	<0.001
Gestational weight gain (kg)	15.8 ± 4.4	12.8 ± 3.6	<0.001
HbA1c (%)	5.77 ± 0.8	5.09 ± 0.39	<0.001
FBG (mg/dL)	102.18 ± 16.07	85.64 ± 11.32	<0.001
Physical activity (MET-h/week)	32.0 ± 8.6	32.2 ± 8.9	0.746
Sleep duration (h/day)	6.92 ± 1.13	6.93 ± 1.07	0.875
Clinical Measurements			
SBP (mmHg)	117.1 ± 11.5	108.3 ± 11.3	<0.001
DBP (mmHg)	76.5 ± 7.6	71.0 ± 6.8	<0.001
Medical History (%)			
Family history of diabetes	47.8	21.5	<0.001
Previous GDM	27.3	9.0	<0.001
PCOS	15.8	17.3	0.568
Smoking	7.0	10.0	0.128
Dietary Index			
DI-GM score	7.09 ± 1.64	9.52 ± 1.83	<0.001

Values are presented as mean ± standard deviation or percentage as appropriate. Between-group comparisons were performed using independent *t*-tests for continuous variables and chi-square tests for categorical variables. BMI, body mass index; SBP, systolic blood pressure; DBP, diastolic blood pressure; MET, metabolic equivalent task; PCOS, polycystic ovary syndrome; GDM, gestational diabetes mellitus.

Compared with controls, GDM cases had significantly higher HbA1c, FBG, insulin, OGTT 1-h and 2-h glucose, IL-6, TNF-α, LPS, and triglycerides, along with lower HDL-C (all *p* < 0.05). C-peptide, LDL-C, total cholesterol, and zonulin did not differ significantly between groups. hs-CRP was significantly higher in GDM cases (3.76 ± 1.30 vs. 3.40 ± 0.98 mg/L, *p* < 0.001), consistent with the expected pro-inflammatory state in GDM (Supplementary Table S1).

Table 2 presents dietary intake and food group composition across DI-GM tertiles among controls and GDM cases. In both groups, increasing DI-GM tertiles were associated with significantly lower energy intake, total fat, carbohydrate, sugar, saturated fat, and ultra-processed food consumption (all *P*-trend<0.001). In contrast, protein, dietary fiber, omega-3 fatty acids, magnesium, vitamin C, and polyphenol intake increased progressively across tertiles. Participants in the highest DI-GM tertile also reported greater consumption of microbiota-supportive foods, including fruits, vegetables, legumes, whole grains, fermented foods, nuts, olive oil, and fish, whereas intake of refined grains, red meat, processed meat, fast food, and sugar-sweetened beverages decreased significantly across tertiles in both controls and GDM cases (all *P*-trend<0.001) (Table 2). Notably, energy intake differences across DI-GM tertiles were modest within both groups—from 2308.2 to 2155.9 kcal/day in controls (~152 kcal/day) and from 2423.1 to 2204.2 kcal/day in GDM cases (~219 kcal/day). While statistically significant due to the large sample size, these differences are modest in absolute terms.

**Table 2:** Dietary intake and food group nutrient composition across DI-GM tertiles in controls and GDM cases.

Variable	Controls T1	Controls T2	Controls T3	*P*-trend	GDM T1	GDM T2	GDM T3	*P*-trend
DI-GM Score	7.52 ± 0.98	9.56 ± 0.47	11.52 ± 0.90	<0.001	5.33 ± 0.93	7.08 ± 0.39	8.88 ± 0.88	<0.001
Dietary Intake								
Energy (kcal/day)	2308.2 ± 336.7	2187.9 ± 362.8	2155.9 ± 381.5	0.004	2423.1 ± 338.3	2312.5 ± 346.9	2204.2 ± 328.4	<0.001
Protein (g/day)	74.9 ± 14.7	79.5 ± 16.5	87.3 ± 12.7	<0.001	72.9 ± 16.1	77.7 ± 16.1	81.1 ± 16.1	0.002
Fat (g/day)	80.9 ± 13.7	70.6 ± 14.2	64.4 ± 15.6	<0.001	92.0 ± 15.1	82.2 ± 14.7	71.3 ± 13.8	<0.001
Carbohydrates (g/day)	304.3 ± 46.4	281.0 ± 48.3	251.7 ± 51.0	<0.001	320.0 ± 46.2	298.2 ± 47.5	272.8 ± 42.1	<0.001
Fiber (g/day)	17.8 ± 6.3	24.7 ± 6.5	30.2 ± 5.6	<0.001	15.9 ± 5.2	22.1 ± 6.1	27.7 ± 6.1	<0.001
Sugar (g/day)	104.8 ± 21.2	81.3 ± 20.5	61.0 ± 17.1	<0.001	119.6 ± 22.2	89.8 ± 18.9	66.1 ± 21.0	<0.001
Saturated fat (g/day)	30.4 ± 7.3	24.3 ± 6.7	16.5 ± 6.7	<0.001	34.0 ± 7.5	27.2 ± 6.8	18.3 ± 7.0	<0.001
Omega-3 (g/day)	1.07 ± 0.49	1.42 ± 0.50	1.86 ± 0.47	<0.001	1.12 ± 0.57	1.21 ± 0.44	1.61 ± 0.47	<0.001
Magnesium (mg/day)	264.5 ± 51.7	333.8 ± 55.2	432.9 ± 59.5	<0.001	260.2 ± 42.2	306.5 ± 51.8	391.1 ± 58.7	<0.001
Vitamin C (mg/day)	77.2 ± 24.6	94.6 ± 30.9	127.7 ± 22.1	<0.001	74.5 ± 28.5	84.1 ± 24.4	112.4 ± 27.3	<0.001
Polyphenols (mg/day)	872.8 ± 251.1	1106.4 ± 258.6	1364.4 ± 261.1	<0.001	800.0 ± 224.8	952.8 ± 230.9	1257.0 ± 260.2	<0.001
Microbiota-Supportive Foods								
Fruit (servings/day)	2.24 ± 0.98	2.81 ± 0.99	3.24 ± 1.07	<0.001	2.15 ± 0.93	2.56 ± 0.86	3.35 ± 1.07	<0.001
Vegetables (servings/day)	2.71 ± 1.16	3.49 ± 1.16	4.56 ± 1.21	<0.001	2.49 ± 1.15	3.11 ± 1.25	3.91 ± 1.16	<0.001
Legumes (servings/week)	2.73 ± 1.09	3.45 ± 1.10	4.22 ± 1.12	<0.001	2.38 ± 0.90	3.17 ± 1.24	4.11 ± 1.07	<0.001
Whole grains (servings/day)	1.75 ± 0.89	2.23 ± 0.89	2.80 ± 0.88	<0.001	1.60 ± 0.90	2.03 ± 0.94	2.64 ± 0.85	<0.001
Fermented foods (servings/week)	1.41 ± 0.68	1.84 ± 0.65	2.26 ± 0.67	<0.001	1.26 ± 0.69	1.56 ± 0.65	2.07 ± 0.74	<0.001
Nuts (g/day)	18.2 ± 7.3	21.9 ± 7.7	25.8 ± 7.5	<0.001	15.6 ± 6.9	20.2 ± 8.1	25.1 ± 8.2	<0.001
Olive oil (g/day)	14.4 ± 5.9	17.3 ± 5.6	22.7 ± 4.7	<0.001	11.7 ± 6.0	16.5 ± 6.0	20.9 ± 5.7	<0.001
Fish (g/day)	7.77 ± 0.51	9.74 ± 0.56	12.69 ± 0.59	<0.001	6.78 ± 0.38	8.57 ± 0.50	11.10 ± 0.51	<0.001
Microbiota-Disruptive Foods								
Refined grains (servings/day)	7.07 ± 1.49	5.52 ± 1.30	4.24 ± 1.77	<0.001	7.57 ± 1.43	6.22 ± 1.44	4.48 ± 1.44	<0.001
Red meat (servings/week)	5.53 ± 1.37	4.62 ± 1.44	3.36 ± 1.59	<0.001	6.48 ± 1.59	4.84 ± 1.38	3.61 ± 1.39	<0.001
Processed meat (servings/week)	2.87 ± 0.91	2.13 ± 0.89	1.87 ± 0.89	<0.001	3.15 ± 1.10	2.49 ± 0.84	1.84 ± 0.85	<0.001
Fast food (servings/week)	2.37 ± 0.76	1.79 ± 0.72	1.39 ± 0.79	<0.001	2.39 ± 0.73	1.98 ± 0.80	1.51 ± 0.72	<0.001
Ultra-processed food (%)	13.85 ± 0.55	10.26 ± 0.57	7.04 ± 0.40	<0.001	15.78 ± 0.56	11.76 ± 0.53	8.34 ± 0.50	<0.001
SSB (servings/day)	1.74 ± 0.67	1.44 ± 0.71	1.12 ± 0.55	<0.001	1.85 ± 0.66	1.64 ± 0.72	1.16 ± 0.60	<0.001

Values are presented as mean ± standard deviation. Participants are stratified into DI-GM tertiles (T1–T3) separately for controls and GDM cases. *P*-trend values were calculated using trend analysis across increasing DI-GM tertiles within each group. Dietary variables include total energy intake, macronutrients, and food group nutrient composition, categorized as microbiota-supportive foods (fruits, vegetables, whole grains, legumes, fermented foods, nuts, and olive oil) and microbiota-disruptive foods (red meat, processed meat, fast food, and sugar-sweetened beverages). Abbreviations:; SSB, sugar-sweetened beverages; GDM, gestational diabetes mellitus.

As shown in Table 3, several glycemic and metabolic biomarkers were associated with DI-GM tertiles, with distinct patterns observed between controls and GDM cases.

**Table 3:** Clinical, metabolic, inflammatory, and gut-derived markers according to DI-GM tertiles in controls and GDM groups.

Variable	Controls T1	Controls T2	Controls T3	*P*-trend	GDM T1	GDM T2	GDM T3	*P*-trend
Glycemic Markers								
HbA1c (%)	5.22 ± 0.42	5.05 ± 0.36	5.08 ± 0.39	0.039	5.85 ± 0.74	5.79 ± 0.67	5.14 ± 1.10	<0.001
Insulin (μIU/mL)	9.97 ± 3.76	9.60 ± 3.63	9.59 ± 3.46	0.797	15.92 ± 5.34	16.30 ± 5.47	15.98 ± 6.11	0.803
HOMA-IR	2.03 ± 0.76	1.90 ± 0.72	2.20 ± 0.75	0.057	3.91 ± 1.33	3.67 ± 1.44	3.33 ± 1.08	0.029
FBG (mg/dL)	85.7 ± 11.7	85.6 ± 11.5	85.7 ± 11.2	0.998	103.5 ± 16.2	101.8 ± 14.8	96.1 ± 18.5	0.033
1-h OGTT glucose (mg/dL)	131.70 ± 11.71	131.60 ± 11.46	131.65 ± 11.22	0.970	183.22 ± 24.34	180.65 ± 22.25	172.15 ± 27.84	0.033
2-h OGTT glucose (mg/dL)	117.69 ± 10.6	117.58 ± 10.5	117.68 ± 11.2	0.960	158.2 ± 24.3	155.6 ± 21.99	147.1 ± 28.0	0.035
C-peptide	9.68 ± 3.01	9.93 ± 2.50	9.92 ± 2.79	0.684	9.35 ± 4.01	10.20 ± 2.81	9.98 ± 3.02	0.272
Lipid Profile								
Triglycerides (mg/dL)	231.00 ± 7.04	185.06 ± 6.96	132.50 ± 7.56	<0.001	278.57 ± 6.88	223.78 ± 7.73	161.93 ± 8.78	<0.001
HDL-C (mg/dL)	41.54 ± 2.09	50.94 ± 1.89	64.89 ± 1.81	<0.001	35.76 ± 2.15	44.17 ± 2.33	56.61 ± 2.13	<0.001
LDL-C (mmol/L)	4.62 ± 1.23	4.6 ± 1.17	4.49 ± 1.94	0.045	4.92 ± 1.52	4.9 ± 1.5	4.88 ± 1.6	0.994
Total Cholesterol (mmol/L)	4.9 ± 1.01	4.1 ± 1.09	4.9 ± 1.01	0.372	5.2 ± 1.23	4.9 ± 1.17	5.1 ± 1.94	0.450
Inflammatory Markers								
hs-CRP (mg/L)	3.87 ± 0.77	2.98 ± 0.84	2.19 ± 0.86	<0.001	5.80 ± 0.73	4.52 ± 0.87	2.93 ± 0.76	<0.001
IL-6 (pg/mL)	3.54 ± 1.44	3.73 ± 1.54	3.82 ± 1.43	0.480	6.65 ± 2.15	6.73 ± 2.20	7.22 ± 2.54	0.349
TNF-α (pg/mL)	11.52 ± 2.86	11.21 ± 2.73	11.44 ± 3.23	0.752	15.55 ± 4.34	16.07 ± 4.33	16.19 ± 3.52	0.436
Adiponectin (μg/mL)	10.76 ± 3.31	10.45 ± 3.18	10.66 ± 2.75	0.763	6.88 ± 2.44	7.01 ± 2.69	7.67 ± 2.92	0.219
Gut-Derived Metabolites								
LPS	10.11 ± 3.30	9.86 ± 2.92	8.17 ± 1.80	0.001	10.08 ± 3.14	9.25 ± 2.97	8.16 ± 1.92	0.001
Zonulin	9.52 ± 2.88	9.66 ± 2.84	10.04 ± 2.92	0.345	9.58 ± 3.13	10.13 ± 2.73	9.79 ± 3.14	0.240

Values are presented as mean ± standard deviation. *P*-trend values were obtained using one-way ANOVA for continuous variables across DI-GM tertiles. Biomarkers include glycemic parameters, lipid profile, inflammatory markers, and gut-derived metabolites. HbA1c, glycated hemoglobin; HOMA-IR, homeostatic model assessment of insulin resistance; FBG, fasting blood glucose; OGTT, oral glucose tolerance test; hs-CRP, high-sensitivity C-reactive protein; LPS, lipopolysaccharide; GDM, gestational diabetes mellitus. T1, first tertile of the Dietary Index for Gut Microbiota (DI-GM) score; T2, second tertile of the DI-GM score; T3, third tertile of the DI-GM score.

Among controls, HbA1c levels showed a modest decreasing trend across tertiles (5.22, 5.05, and 5.08%; *P*-trend = 0.039), although the pattern was not strictly monotonic, while HOMA-IR did not show a significant linear trend (*p* = 0.057), with values of 2.03, 1.90, and 2.20 across T1 to T3. Fasting blood glucose and OGTT glucose concentrations did not differ substantially across tertiles in controls. Triglyceride concentrations were significantly lower across tertiles (231.0 ± 7.0, 185.1 ± 7.0, and 132.5 ± 7.6 mg/dL; *P*-trend < 0.001), whereas HDL-C levels were significantly higher (41.5 ± 2.1, 50.9 ± 1.9, and 64.9 ± 1.8 mg/dL; *P*-trend < 0.001). hs-CRP concentrations also decreased across tertiles (3.87 ± 0.77, 2.98 ± 0.84, and 2.19 ± 0.86 mg/L; *P*-trend < 0.001), and LPS concentrations showed a decreasing trend. No significant trends were observed for insulin, IL-6, TNF-α, adiponectin, and zonulin concentrations among controls.

Among GDM cases, higher DI-GM tertiles were associated with lower HbA1c levels (5.85 ± 0.74, 5.79 ± 0.67, 5.14 ± 1.10%; *P*-trend < 0.001), HOMA-IR (3.91 ± 1.33, 3.67 ± 1.44, 3.33 ± 1.08; *P*-trend = 0.029), fasting glucose (103.5, 101.8, 96.1 mg/dL; *P*-trend = 0.033), and OGTT 1-h and 2-h glucose values. Triglyceride concentrations were substantially lower across tertiles (278.6, 223.8, and 161.9 mg/dL; *P*-trend<0.001), whereas HDL-C levels were significantly higher (35.8, 44.2, and 56.6 mg/dL; *P*-trend < 0.001). hs-CRP decreased across tertiles (5.80 ± 0.73, 4.52 ± 0.87, and 2.93 ± 0.76 mg/L; *P*-trend < 0.001), and LPS concentrations showed a decreasing trend. No significant trends were observed for insulin, IL-6, TNF-α, adiponectin, and zonulin concentrations in GDM cases.

Overall, triglyceride and hs-CRP reductions, as well as HDL-C increases, were observed across DI-GM tertiles in both groups, with larger absolute changes in the GDM group. Notably, LPS concentrations decreased across tertiles in both controls (*P*-trend = 0.001) and GDM cases (*P*-trend = 0.001), suggesting that greater adherence to the DI-GM dietary pattern is associated with lower circulating endotoxin levels regardless of glycemic status.

Table 4 summarizes maternal and neonatal outcomes across DI-GM tertiles. In the control group, higher DI-GM adherence was only associated with a significantly higher rate of cesarean delivery (*P*-trend < 0.001), while no significant trends were observed for the other outcomes (all *P*-trend > 0.10). Among women with GDM, strong inverse associations were observed for all outcomes, with highly significant trends (all *P*-trend < 0.001). Notably, in GDM cases, cesarean delivery decreased from all cases in T1 (47/47) to 16.1% in T3, preterm birth from 74.5 to 6.1%, and macrosomia from 53.2 to 4.8% (all *P*-trend < 0.001). No significant trends were observed for APGAR scores.

**Table 4:** Maternal and neonatal outcomes across DI-GM tertiles stratified by GDM status.

Variable	Control T1	Control T2	Control T3	*P*-trend	GDM T1	GDM T2	GDM T3	*P*-trend
Delivery Mode								
Cesarean delivery, *n* (%)	28 (12.7)	22 (15.4)	16 (43.2)	**<0.001**	47 (100.0)	49 (39.8)	37 (16.1)	<0.001
Pregnancy Outcomes								
Preterm birth, *n* (%)	19 (8.6)	13 (9.1)	7 (18.9)	0.135	35 (74.5)	24 (19.5)	14 (6.1)	<0.001
Gestational hypertension, *n* (%)	16 (7.3)	11 (7.7)	6 (16.2)	0.160	31 (66.0)	22 (17.9)	14 (6.1)	<0.001
Preeclampsia, *n* (%)	11 (5.0)	7 (4.9)	3 (8.1)	0.584	24 (51.1)	17 (13.8)	10 (4.3)	<0.001
Neonatal Outcomes								
Neonatal hypoglycemia, *n* (%)	9 (4.1)	6 (4.2)	3 (8.1)	0.413	28 (59.6)	20 (16.3)	12 (5.2)	<0.001
Macrosomia, *n* (%)	8 (3.6)	5 (3.5)	2 (5.4)	0.731	25 (53.2)	18 (14.6)	11 (4.8)	<0.001
NICU admission, *n* (%)	10 (4.5)	7 (4.9)	4 (10.8)	0.220	27 (57.4)	21 (17.1)	13 (5.7)	<0.001
Neonatal jaundice, *n* (%)	12 (5.5)	8 (5.6)	4 (10.8)	0.341	29 (61.7)	22 (17.9)	15 (6.5)	<0.001
Respiratory distress, *n* (%)	7 (3.2)	5 (3.5)	2 (5.4)	0.562	22 (46.8)	16 (13.0)	10 (4.3)	<0.001
APGAR1	8.85 ± 2.92	8.96 ± 3.04	9.52 ± 3.36	0.451	8.12 ± 3.04	8.58 ± 2.83	9.14 ± 2.71	0.039
APGAR5	9.36 ± 2.99	9.37 ± 2.62	9.92 ± 3.19	0.533	8.64 ± 2.95	9.08 ± 2.81	9.74 ± 2.66	0.027
GWG (kg)	12.84 ± 3.86	13.32 ± 3.57	12.5 ± 3.6	0.214	15.8 ± 4.4	15.8 ± 4.51	15.9 ± 4.1	0.992

Values are presented as mean ± standard deviation or number/percentage as appropriate. *P*-trend values were calculated using one-way ANOVA for continuous variables and chi-square tests for categorical variables across DI-GM tertiles. Higher DI-GM adherence was associated with significantly lower prevalence of adverse maternal and neonatal outcomes among women with gestational diabetes mellitus, particularly neonatal hypoglycemia, macrosomia, preeclampsia, gestational hypertension, and NICU admission. Data extracted from uploaded cross-tabulation and ANOVA outputs.

Table 5 presents the multivariable logistic regression analyses examining the association between DI-GM tertiles and GDM risk. Compared with participants in the lowest tertile (T1), those in the middle (T2) and highest tertiles (T3) had progressively lower odds of GDM in crude analyses (T2: OR = 0.25, 95% CI: 0.17–0.38; T3: OR = 0.20, 95% CI: 0.15–0.40). After adjustment for maternal age, pre-pregnancy BMI, energy intake, physical activity, sleep duration, family history of diabetes, and gestational weight gain (Model 1), the inverse association remained statistically significant for T3 but was attenuated for T2. Further adjustment for prenatal antibiotic exposure and probiotic supplementation (Model 2) produced similar results. In the fully adjusted model, participants in T3 had 55.4% lower odds of GDM compared with those in T1 (OR = 0.446, 95% CI: 0.269–0.738), whereas the association for T2 remained non-significant (OR = 0.819, 95% CI: 0.537–1.252). A significant dose–response relationship was observed across increasing DI-GM tertiles (*P*-trend = 0.002). Furthermore, each 1-unit increase in DI-GM score was associated with an 11.0% reduction in the adjusted odds of GDM (OR = 0.890, 95% CI: 0.811 0.977). However, the non-significant association observed in the middle tertile (T2) alongside the significant association in the highest tertile (T3) suggests that the relationship may not be strictly linear across the entire DI-GM range. The continuous model estimates the average association across the full dietary score distribution, whereas categorical analyses compare broader exposure groups. Collectively, these findings are compatible with a threshold effect, whereby clinically meaningful reductions in GDM odds become evident primarily among women achieving the highest adherence to a microbiota oriented dietary pattern. Nevertheless, the significant trend across tertiles (*P* trend = 0.002) supports an overall inverse dose–response relationship. The per-unit OR should be interpreted as an average effect across the observed score range rather than as implying identical risk reduction for every incremental increase.

**Table 5:** Multivariable logistic regression for GDM risk across DI-GM tertiles.

DI-GM tertile	Crude OR (95% CI)	Model 1 OR (95% CI)	Model 2 OR (95% CI)
T1 (Lowest)	1.00 (Reference)	1.00 (Reference)	1.00 (Reference)
T2 (Middle)	0.25 (0.17–0.38)	0.825 (0.541–1.258)	0.819 (0.537–1.252)
T3 (Highest)	0.20 (0.15–0.40)	0.448 (0.271–0.741)	0.446 (0.269–0.738)
*P* for trend	<0.001	0.002	0.002
Per 1-unit increase in DI-GM	0.45 (0.40–0.50)	0.892 (0.813–0.978)	0.890 (0.811–0.977)

Odds ratios (Ors) with 95% confidence intervals (Cis) are presented.

Model 1: Adjusted for age, pre-pregnancy BMI, energy intake, physical activity, sleep duration, family history of diabetes, and gestational weight gain.

Model 2: Adjusted for Model 1 covariates plus prenatal antibiotic exposure and probiotic exposure.

*P* for trend was calculated using DI-GM tertiles as an ordinal variable.

GDM, gestational diabetes mellitus; DI-GM, Gut Microbiota-Oriented Dietary Index; BMI, body mass index.

**Figure 2:**
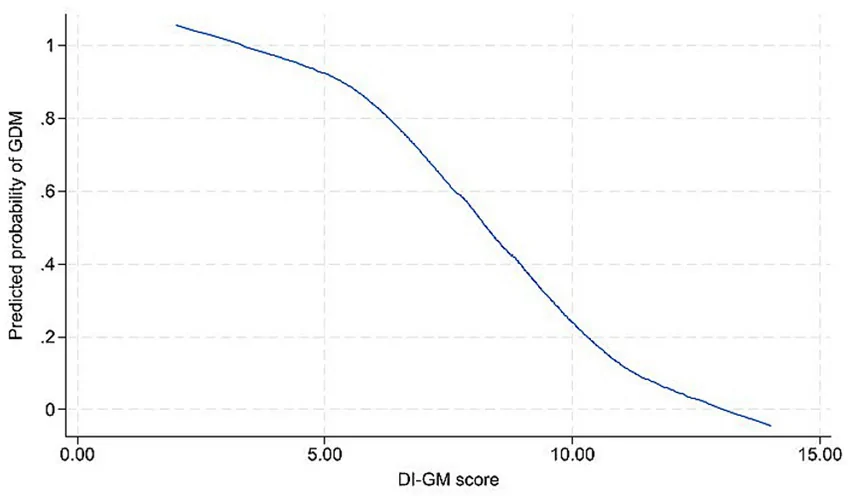
Predicted probability of GDM by DI-GM score. Higher DI-GM scores were associated with a significantly lower predicted probability of GDM (*P-trend* <0.001), indicating an inverse dose–response relationship.

Figure 2 illustrates the inverse dose–response association between DI-GM score and the predicted probability of GDM, demonstrating a progressive decline in estimated GDM risk with increasing adherence to a gut microbiota-supportive dietary pattern.

To assess the robustness of our findings and address concerns regarding residual confounding, we performed extensive sensitivity analyses (Supplementary Table S2). The inverse association between DI-GM and GDM remained consistent across all models. In the main adjusted analysis (*n* = 800), each 1-unit increase in DI-GM was associated with 58% lower odds of GDM (OR = 0.42, 95% CI: 0.36–0.48; *β* = −0.870, *p* < 0.001). After excluding participants with a family history of diabetes (*n* = 523), the association remained virtually unchanged (OR = 0.43, 95% CI: 0.36–0.51; *β* = −0.845, *p* < 0.001). Stratified analyses by BMI categories confirmed the inverse association across all strata. The strongest association was observed among women with pre-pregnancy obesity (T3 vs. T1: OR = 0.008, 95% CI: 0.002–0.032, *p* < 0.001). However, because the obese subgroup represented a relatively small proportion of the study population, this estimate should be interpreted cautiously and may reflect greater statistical instability than observed in the overall cohort. Additional adjustment for dietary confounders including fiber, saturated fat, sugar, and ultra-processed food consumption did not materially attenuate the association (OR = 0.42, 95% CI: 0.36–0.49). Bootstrap validation with 1,000 replications confirmed the stability of the estimates (bias-corrected 95% CI: 0.36–0.49). The *E*-value for the per-unit OR was 3.98 (95% CI: 3.41), indicating that an unmeasured confounder would need to be associated with both DI-GM and GDM with a risk ratio of 3.98 each to fully explain the observed association—a magnitude substantially larger than most established risk factors. These sensitivity analyses consistently provide additional support for the robustness of our findings against residual confounding, selection bias, and model specification concerns.

Adjusted analyses (Supplementary Table S3) showed that higher DI-GM adherence was associated with significantly lower odds of adverse maternal and neonatal outcomes among women with GDM, including preterm birth (OR = 0.33, 95% CI: 0.18–0.61), gestational hypertension (OR = 0.39, 95% CI: 0.20–0.75), preeclampsia (OR = 0.38, 95% CI: 0.17–0.82), neonatal hypoglycemia (OR = 0.36, 95% CI: 0.18–0.73), macrosomia (OR = 0.38, 95% CI: 0.18–0.79), NICU admission (OR = 0.42, 95% CI: 0.21–0.82), and respiratory distress (OR = 0.40, 95% CI: 0.18–0.87). Similar patterns were observed among controls, although some confidence intervals included the null due to lower event rates.

The mediation analysis findings are presented in Table 6. Higher DI-GM scores demonstrated significant direct inverse associations with all evaluated glycemic outcomes, including HOMA-IR (*β* = −0.257, 95% CI: −0.305 to −0.209, *p* < 0.001), fasting blood glucose (*β* = −2.642, 95% CI: −3.209 to −2.076, *p* < 0.001), and HbA1c (*β* = −0.125, 95% CI: −0.150 to −0.099, *p* < 0.001). Significant indirect effects through hs-CRP were also identified for HOMA-IR (*β* = −0.065, 95% CI: −0.088 to −0.042, *p* < 0.001), FBG (*β* = −0.549, 95% CI: −0.810 to −0.289, *p* < 0.001), and HbA1c (*β* = −0.021, 95% CI: −0.033 to −0.009, *p* < 0.001). Notably, these indirect effects were negative, indicating an inconsistent (competitive) mediation pattern in which the protective direct effect of DI-GM was partially offset by the pathway through hs-CRP. This statistical phenomenon may reflect the complexity of interconnected inflammatory and metabolic pathways, shared underlying processes, residual confounding, or concurrent regulation of inflammation and glucose metabolism, rather than a biologically adverse role of hs-CRP. Although the indirect effects attenuated the magnitude of the associations, the total effects of DI-GM on HOMA-IR (*β* = −0.192, 95% CI: −0.236 to −0.148), FBG (*β* = −2.093, 95% CI: −2.608 to −1.578), and HbA1c (*β* = −0.104, 95% CI: −0.127 to −0.081) remained statistically significant (all *p* < 0.001). Given the concurrent measurement of all biomarkers at 24–28 weeks, temporal precedence cannot be established, and causal mediation cannot be inferred. Consequently, these findings should be interpreted as exploratory and hypothesis-generating, requiring confirmation in prospective longitudinal studies.

**Table 6:** Mediation analysis: effect of DI-GM on glycemic outcomes through hs-CRP.

Outcome variable	Path	Effect type	*β* (95% CI)	*P*-value
HOMA-IR	DI-GM → HOMA-IR	Direct Effect	−0.257 (−0.305, −0.209)	<0.001
	DI-GM → hs-CRP → HOMA-IR	Indirect Effect	−0.065 (−0.088, −0.042)	<0.001
		Total Effect	−0.192 (−0.236, −0.148)	<0.001
FBG (mg/Dl)	DI-GM → FBG	Direct Effect	−2.642 (−3.209, −2.076)	<0.001
	DI-GM → hs-CRP → FBG	Indirect Effect	−0.549 (−0.810, −0.289)	<0.001
		Total Effect	−2.093 (−2.608, −1.578)	<0.001
HbA1c (%)	DI-GM → HbA1c	Direct Effect	−0.125 (−0.150, −0.099)	<0.001
	DI-GM → hs-CRP → HbA1c	Indirect Effect	−0.021 (−0.033, −0.009)	<0.001
		Total Effect	−0.104 (−0.127, −0.081)	<0.001

Structural equation modeling (SEM) was performed using maximum likelihood estimation. Models were adjusted for age, pre-pregnancy body mass index (PreBMI), energy intake, parity, physical activity, sleep duration, family history of diabetes, and gestational weight gain (GWG). Indirect, direct, and total effects were estimated using estat teffects after SEM. hs-CRP, high-sensitivity C-reactive protein; HOMA-IR, homeostatic model assessment of insulin resistance; FBG, fasting blood glucose; HbA1c, glycated hemoglobin.

## Discussion

To our knowledge, this is among the largest case–control studies evaluating DI-GM scores in relation to maternal metabolic and neonatal outcomes. Greater DI-GM adherence was associated with substantially lower GDM odds, more favorable glycemic and lipid profiles, lower systemic inflammation, and improved maternal and neonatal outcomes. Women with GDM exhibited significantly lower DI-GM scores than controls. Higher DI-GM adherence correlated with lower fasting glucose, HbA1c, HOMA-IR, triglycerides, hs-CRP, and LPS, alongside higher HDL-C. Mediation analysis suggested hs-CRP may partially mediate the DI-GM-glycemic relationship, though this requires prospective confirmation. The inverse DI-GM-LPS association observed in both GDM cases and controls suggests that microbiota-supportive dietary patterns may influence gut barrier function and reduce endotoxemia across the full spectrum of glucose tolerance during pregnancy. However, as an observational case–control study, these findings represent associations, not causality. Gut microbiota was inferred, not directly measured, and requires confirmation in prospective studies.

The inverse association between DI-GM adherence and GDM risk is biologically plausible and consistent with previous reports linking dietary patterns to GDM risk (18, 19, 25, 28). For example, higher adherence to the Phytochemical Dietary Index (PDI), Mediterranean diet score, and Dietary Inflammatory Index (DII)-based healthy patterns has been associated with lower odds of GDM, with effect estimates generally ranging from approximately 0.60 to 0.85 across higher adherence categories compared with lowest intake groups in observational studies (18, 19, 25, 28). Diets rich in fruits, vegetables, legumes, whole grains, nuts, fermented foods, fish, and olive oil provide substantial amounts of dietary fiber, polyphenols, omega-3 fatty acids, antioxidants, and micronutrients that may improve insulin sensitivity and metabolic homeostasis (14, 16, 18). In contrast, microbiota-disruptive dietary components including refined carbohydrates, processed meat, ultra-processed foods, saturated fat, and sugar-sweetened beverages may contribute to chronic inflammation, oxidative stress, gut dysbiosis, and impaired insulin signaling (28, 29). However, gut microbiota was not directly measured; thus, the proposed microbiota-related mechanisms remain inferential and require confirmation through metagenomics profiling in future studies. Importantly, the pattern of association in our study warrants careful interpretation. Although a significant trend was observed across tertiles (*P*-trend = 0.002), the non-significant association in the middle tertile (T2) alongside the significant protective effect in the highest tertile (T3) suggests a non-linear relationship, potentially reflecting a threshold effect whereby meaningful changes in gut microbiota composition, systemic inflammation, and metabolic regulation require a critical level of dietary modification (14, 16). Such threshold effects are common in nutritional epidemiology, particularly with indices capturing multiple interacting components (21–23). The T2 range may not represent sufficient deviation from typical dietary patterns to confer measurable metabolic benefits, whereas T3 reflects a qualitatively different dietary profile that substantially promotes gut microbial health and reduces inflammatory burden. Mean DI-GM scores observed in the present study were broadly comparable to those reported in previous studies using the original DI-GM framework, although direct comparison should be interpreted cautiously because dietary assessment methods, population characteristics, and cohort-specific scoring distributions differed across studies. The magnitude of the observed associations warrants careful consideration. Our extensive sensitivity analyses—including stratified analyses, exclusion of high-risk groups, additional dietary confounding adjustment, bootstrap validation, and E-value assessment—consistently supported the robustness of the findings. The *E*-value analysis (3.98) suggests that substantial unmeasured confounding would be required to negate the observed associations. Residual confounding cannot be completely excluded, but the consistency across multiple sensitivity analyses strengthens confidence in the validity of our findings.

Several observed associations warrant explicit consideration. The OR of 0.008 (95% CI: 0.002–0.032) for highest DI-GM adherence among obese women, while statistically robust, represents an unusually large effect estimate, potentially explained by the high-risk nature of this subgroup, the cumulative effect of multiple dietary dimensions, the case–control design, or residual confounding. Strong correlations between DI-GM and lipid parameters (*r* = 0.749 with triglycerides, *r* = 0.780 with HDL-C) reflect the inclusion of dietary fat and fiber components linked to lipid metabolism. While sensitivity analyses (*E*-value = 3.98) support robustness, these findings require confirmation in prospective cohorts and RCTs. Methodologically, DI-GM cut-offs derived from cohort-specific medians reflect relative adherence rather than an absolute benchmark, warranting caution when comparing scores across populations. Clinically, modest dietary improvements may be insufficient to reduce GDM risk; more substantial dietary changes may be necessary. The per-unit OR (0.890) represents an average effect, not a uniform linear effect, and future studies using flexible dose–response modeling are needed to characterize non-linear associations and identify optimal dietary targets.

One of the major findings of the present study was the progressive reduction in inflammatory biomarkers, particularly hs-CRP, across increasing DI-GM tertiles. Overall biomarker comparisons (Supplementary Table S1) showed significantly higher glycemic parameters, IL-6, TNF-α, hs-CRP, LPS, and triglycerides in GDM cases, alongside lower HDL-C, consistent with established GDM pathophysiology (7, 28). These comparisons provide context for the tertile analyses in Tables 3, 4. Chronic low-grade inflammation is recognized as a central mechanism in GDM pathogenesis (30). Pregnancy itself induces physiological insulin resistance; however, exaggerated inflammatory activation may impair insulin receptor signaling and glucose uptake in peripheral tissues (4, 7). Adipose tissue-derived inflammatory cytokines such as IL-6 and TNF-α may activate inflammatory cascades including nuclear factor-kappa B (NF-κB) signaling pathways, leading to worsening insulin resistance and β-cell dysfunction (31). The observed reduction in hs-CRP among participants with higher DI-GM adherence suggests that dietary patterns captured by the DI-GM may be associated with lower systemic inflammatory burden during pregnancy. Importantly, our study assessed a microbiota oriented dietary index and related circulating biomarkers (LPS, zonulin) as proxies for gut microbial ecology. Direct fecal microbiome sequencing was not performed. Therefore, the inferred gut microbiota mechanisms should be considered hypothesis generating, and the observed associations between DI-GM adherence and biomarkers of gut permeability and endotoxemia should be interpreted as indirect evidence of potential microbial involvement. These findings support the potential value of microbiota-oriented dietary strategies as a complementary approach to GDM prevention, particularly among women with pre-pregnancy obesity or a family history of diabetes (1, 4, 5, 20–23). Further prospective and interventional studies are required to establish causality and evaluate clinical implementation. Several biomarkers central to the proposed mechanisms, including IL-6, TNF-α, and adiponectin, did not differ significantly across DI-GM tertiles. These null findings may reflect that IL-6 and TNF-α are more responsive to acute stimuli than habitual diet, whereas hs-CRP is a more stable marker of chronic inflammation. Collectively, these findings highlight the complexity of the diet–inflammation–metabolism axis, suggesting that associations may not be expressed uniformly across all pathways. Accordingly, proposed mechanisms should be interpreted with caution and require confirmation in prospective studies incorporating direct microbiome measurements.

The mediation analysis provides exploratory support for this hypothesis, showing significant indirect effects of DI-GM on glycemic outcomes through hs-CRP. Although partial, these effects support the concept that anti-inflammatory dietary mechanisms may contribute to improved glycemic regulation. However, the negative indirect effects indicate an inconsistent (competitive) mediation pattern, where the protective direct effect was partially offset by the pathway through hs-CRP—a finding biologically unexpected if hs-CRP functioned as a classical mediator. Several explanations may account for this: concurrent measurement of all biomarkers at 24–28 weeks precludes temporal precedence (32); the negative indirect effect may reflect residual confounding or statistical non-mediation rather than true biological mediation; hs-CRP is a downstream acute-phase reactant rather than a direct measure of gut microbiota-mediated inflammatory activation (31); and the complex diet-inflammation-glycemia relationship may involve multiple pathways not captured by simple mediation models (20, 22). Direct microbiota-mediated pathways could not be evaluated as gut microbiota composition was not measured (9). Consequently, these mediation findings are exploratory and hypothesis-generating, requiring confirmation in prospective studies with longitudinal measurements and direct gut microbiota characterization (10, 12, 20).

Another notable finding was the association between higher DI-GM adherence and lower LPS concentrations among women with GDM. Elevated circulating LPS reflects metabolic endotoxemia and increased intestinal permeability, both of which have been implicated in obesity-related insulin resistance and metabolic dysfunction (32). Diet-induced gut dysbiosis may impair intestinal barrier integrity, allowing translocation of bacterial endotoxins into systemic circulation and activation of inflammatory pathways (12). Foods emphasized in the DI-GM, including fiber-rich and polyphenol-rich foods, have previously been associated with beneficial microbial activity and gut barrier function, thereby strengthening gut barrier function and reducing endotoxemia (33).

Energy intake decreased modestly across DI-GM tertiles within both groups—approximately 152 kcal/day in controls and 219 kcal/day in GDM cases. While statistically significant, these differences were modest, and adjustment for total energy intake did not materially attenuate the inverse DI-GM-GDM association, nor did additional adjustment for dietary fiber, saturated fat, sugar, or ultra-processed foods. These findings indicate that the protective effect is independent of caloric intake and that dietary quality—rather than energy restriction—is key to metabolic health during pregnancy (14, 16, 18). The favorable lipid profile observed among participants with higher DI-GM adherence—higher HDL-C and lower triglyceride concentrations—further supports this interpretation, as these improvements reflect enhanced insulin sensitivity and reduced hepatic lipogenesis independent of total energy consumption. Diets rich in unsaturated fatty acids, fiber, and antioxidant compounds modulate lipid metabolism through regulation of hepatic lipid synthesis, bile acid metabolism, and inflammatory signaling pathways (34). These observations reinforce that dietary quality, as captured by the DI-GM, confers metabolic benefits beyond simple caloric restriction.

Importantly, higher DI-GM adherence was also associated with more favorable maternal and neonatal outcomes, particularly among women with GDM. Lower frequencies of gestational hypertension, preeclampsia, neonatal hypoglycemia, macrosomia, NICU admission, respiratory distress syndrome, and neonatal jaundice were observed across increasing DI-GM tertiles. These findings may be explained by improved maternal glycemic control, lower inflammatory burden, and better metabolic regulation during pregnancy. Maternal hyperglycemia contributes to excessive fetal glucose exposure, fetal hyperinsulinemia, and abnormal fetal growth, increasing the risk of macrosomia and neonatal metabolic complications. Therefore, improved maternal metabolic status associated with higher DI-GM adherence may contribute to improved neonatal outcomes. Future randomized controlled trials are warranted to determine whether dietary interventions targeting the DI-GM pattern can reduce the incidence of GDM.

The DI-GM should be viewed as complementary to existing dietary quality indices rather than a replacement for them. Several established dietary indices, including the Mediterranean Diet Score, Dietary Approaches to Stop Hypertension (DASH), Dietary Inflammatory Index (DII), Dietary Diversity Score (DDS), and Chinese Healthy Eating Index (CHEI), have been associated with favorable metabolic and pregnancy outcomes (17–19, 25, 28). However, these indices were primarily developed to assess overall dietary quality, dietary diversity, cardiovascular health, or inflammatory potential (17, 18, 25). In contrast, the DI-GM was specifically designed to capture dietary exposures linked to gut microbial composition, gut barrier integrity, microbial metabolite production, and inflammation-related pathways (20–23). Consequently, the DI-GM may provide complementary information regarding the diet–gut microbiota–metabolism axis that is not explicitly incorporated into traditional dietary indices. Future studies should directly compare the predictive performance of DI-GM with established dietary indices, including DASH, DII, Mediterranean Diet Score, DDS, and CHEI, in diverse pregnancy cohorts to determine their relative utility for identifying women at risk of GDM (17–19, 25). However, direct statistical comparison of predictive accuracy between DI-GM and these established indices was not performed in the present study and should be addressed in future validation research. From a clinical and public health perspective, our findings suggest that greater adherence to a gut microbiota-supportive dietary pattern may represent a practical strategy for reducing metabolic risk during pregnancy. Specifically, increased consumption of whole grains, legumes, soy products, fermented dairy foods, dietary fiber-rich foods, and polyphenol-rich foods and beverages such as green tea and coffee, together with reduced intake of refined grains, processed meat, red meat, and high-fat foods, may contribute to improved glycemic control and a lower risk of GDM through mechanisms that may involve inflammatory pathways and potentially gut microbiota-related processes, however, direct microbiota-mediated pathways could not be evaluated because gut microbiota composition was not measured (9–16, 20–23). Although the cohort-specific nature of the DI-GM precludes the establishment of universal clinical cut-off values, women with higher DI-GM scores consistently demonstrated more favorable metabolic profiles and pregnancy outcomes.

This study has several strengths, including comprehensive evaluation of the DI-GM index with inflammatory biomarkers, glycemic and lipid parameters, and maternal-neonatal outcomes within a single framework; a relatively large sample size enabling stratified analyses; detailed adjustment for multiple confounders; and mediation analysis providing mechanistic insight. However, several limitations should be considered. The case–control design precludes causal inference. Dietary intake was assessed via self-reported FFQs, subject to recall bias and measurement error, and residual confounding cannot be excluded. The DI-GM, adapted from a previously published index, has not been formally validated in pregnant populations, and cohort-specific cut-offs limit external validity. Antibiotic and probiotic use were assessed only as binary variables (yes/no) without detailed information on type, dosage, or duration, which may have limited our ability to fully adjust for their effects on gut microbiota composition. Mediation effects were partial, and concurrent biomarker measurement at 24–28 weeks precludes causal mediation inference; these findings are exploratory and hypothesis-generating. Gut microbiota was inferred indirectly rather than directly measured. Several observed associations were larger than commonly reported in nutritional epidemiology, and the magnitude should be interpreted cautiously until confirmed in prospective cohorts and RCTs incorporating metagenomic profiling.

## Conclusion

Greater adherence to a microbiota oriented dietary pattern as captured by the DI-GM index was associated with substantially lower odds of GDM and improved maternal neonatal outcomes in this case control study. However, causality cannot be inferred from this observational design. Furthermore, as direct gut microbiota measurements were not performed, the proposed diet microbiota inflammation pathways remain speculative and require confirmation in prospective studies incorporating metagenomics profiling and randomized controlled trials. The observed associations, which were partly mediated by reduced systemic inflammation in exploratory analyses, warrant further investigation to establish causal mechanisms and clinical applications.

## Data Availability

The raw data supporting the conclusions of this article will be made available by the authors, without undue reservation.
