# Supplementary material for: Dietary index for gut microbiota and odds of gestational diabetes mellitus: associations with inflammatory and metabolic biomarkers
Source: Front Nutr. 2026 Aug 10;13:1895387. doi: 10.3389/fnut.2026.1895387 (PMC13500285; doi:10.3389/fnut.2026.1895387)
Supplement: Supplementary file 1 [file Table_1.DOCX]

**Table S1. Comparison of Biochemical Biomarkers Between GDM Cases and Controls**

| **Biomarker** | **Controls (n=400)** | **GDM Cases (n=400)** | **P-value** |
| --- | --- | --- | --- |
| **Glycemic markers** |  |  |  |
| HbA1c (%) | 5.09 ± 0.39 | 5.77 ± 0.08 | <0.001 |
| FBG (mg/dL) | 85.8 ± 11.32 | 103.1 ± 16.1 | <0.001 |
| Insulin (μIU/mL) | 9.64 ± 3.54 | 16.06 ± 5.45 | <0.001 |
| OGTT 1-h (mg/dL) | 131.64 ± 11.32 | 181.28 ± 24.11 | <0.001 |
| OGTT 2-h (mg/dL) | 117.64 ± 11.32 | 156.28 ± 24.11 | <0.001 |
| C-peptide | 9.79 ± 2.81 | 9.97 ± 3.09 | 0.389 |
| **Lipid profile** |  |  |  |
| Triglycerides (mg/dL) | 194.66 ± 42.15 | 205.47 ± 32.44 | <0.001 |
| HDL-C (mg/dL) | 50.33 ± 8.01 | 47.06 ± 7.46 | <0.001 |
| LDL-C (mmol/L) | 4.91 ± 1.59 | 4.90 ± 1.54 | 0.884 |
| Total cholesterol (mmol/L) | 5.00 ± 1.53 | 5.00 ± 1.53 | 0.951 |
| **Inflammatory markers** |  |  |  |
| hs-CRP (mg/L) | 3.40 ± 0.98 | 3.76 ± 1.30 | <0.001 |
| IL-6 (pg/mL) | 3.76 ± 1.46 | 6.73 ± 2.21 | <0.001 |
| TNF-α (pg/mL) | 11.38 ± 3.03 | 15.80 ± 4.26 | <0.001 |
| **Gut-derived metabolites** |  |  |  |
| LPS | 8.92 ± 3.02 | 9.61 ± 3.11 | 0.002 |
| Zonulin | 9.80 ± 3.00 | 9.86 ± 2.89 | 0.745 |
| **Oxidative stress markers** |  |  |  |
| TAC | 10.23 ± 2.97 | 9.99 ± 3.05 | 0.249 |
| MDA | 9.73 ± 2.95 | 10.18 ± 3.03 | 0.035 |

*Values are presented as mean ± standard deviation. P-values were calculated using independent samples t-tests. Abbreviations: FBG, fasting blood glucose; HOMA-IR, homeostatic model assessment of insulin resistance; OGTT, oral glucose tolerance test; HDL-C, high-density lipoprotein cholesterol; LDL-C, low-density lipoprotein cholesterol; hs-CRP, high-sensitivity C-reactive protein; IL-6, interleukin-6; TNF-α, tumor necrosis factor-alpha; LPS, lipopolysaccharide; TAC, total antioxidant capacity; MDA, malondialdehyde; SOD, superoxide dismutase; GPx, glutathione peroxidase; AST, aspartate aminotransferase; ALT, alanine aminotransferase; ALP, alkaline phosphatase; GGT, gamma-glutamyl transferase.*

**Table S2. Sensitivity Analyses for the Association Between DI-GM and GDM Risk**

| **Sensitivity Analysis** | **n** | **β Coefficient (SE)** | **OR (95% CI)** | **P-value** |
| --- | --- | --- | --- | --- |
| **Main Adjusted Model** | 800 | -0.870 (0.071) | **0.42 (0.36–0.48)** | <0.001 |
| **Excluding Family History of Diabetes** | 523 | -0.845 (0.086) | **0.43 (0.36–0.51)** | <0.001 |
| **Stratified by BMI** |  |  |  |  |
| ─ Normal BMI (<25 kg/m²)* | 155 | — | **0.035 (0.009–0.145)** | <0.001 |
| ─ Overweight (25–29.9 kg/m²)* | 392 | — | **0.027 (0.013–0.059)** | <0.001 |
| ─ Obese (≥30 kg/m²)* | 253 | — | **0.008 (0.002–0.032)** | <0.001 |
| **Additional Dietary Adjustment†** | 800 | — | **0.42 (0.36–0.49)** | <0.001 |
| **Bootstrap Validation (1,000 reps)** | 800 | — | **0.42 (0.36–0.49)** | <0.001 |
| **E-value** | — | — | **3.98 (CI: 3.41)** | — |

*Results shown for T3 vs T1 (highest vs lowest tertile).
†Adjusted for Fiber, SaturatedFat, Sugar, and UPF in addition to main confounders.
Abbreviations: OR, odds ratio; CI, confidence interval; SE, standard error; GDM, gestational diabetes mellitus; BMI, body mass index; UPF, ultra-processed foods.

**Table S3. Adjusted Odds Ratios (95% CI) for Maternal and Neonatal Outcomes by DI-GM Tertile (T3 vs T1)**

| **Outcome** | **Controls** | **GDM Cases** |
| --- | --- | --- |
| **Preterm Birth** | 0.38 (0.15–0.96) | 0.33 (0.18–0.61) |
| **Gestational Hypertension** | 0.36 (0.13–0.99) | 0.39 (0.20–0.75) |
| **Preeclampsia** | 0.26 (0.07–0.94) | 0.38 (0.17–0.82) |
| **Neonatal Hypoglycemia** | 0.31 (0.08–1.16) | 0.36 (0.18–0.73) |
| **Macrosomia** | 0.25 (0.05–1.18) | 0.38 (0.18–0.79) |
| **NICU Admission** | 0.38 (0.12–1.24) | 0.42 (0.21–0.82) |
| **Respiratory Distress** | 0.28 (0.06–1.34) | 0.40 (0.18–0.87) |

*Adjusted for age, pre-pregnancy BMI, energy intake, physical activity, sleep duration, family history of diabetes, and gestational weight gain. Parity was excluded as requested. T3 = highest DI-GM tertile; T1 = reference group.*

**Table S4. Components of the Dietary Index for Gut Microbiota (DI-GM), Scoring**

| **DI-GM Component** | **Category** | **Biological Rationale** | **Median Intake in Study Population (n=800)** | **Primary Scoring Rule** | **Alternative Quartile Scoring (Sensitivity Analysis 1)** | **Alternative Z-score Scoring (Sensitivity Analysis 2)** |
| --- | --- | --- | --- | --- | --- | --- |
| Broccoli | Beneficial | Rich in glucosinolates and fermentable fiber; promotes beneficial microbial taxa | 65 g/day | ≥ Median = 1; < Median = 0 | Q1=0, Q2=1, Q3=2, Q4=3 | Standardized z-score |
| Avocado | Beneficial | Source of monounsaturated fats and fiber associated with microbial diversity | 8 g/day | ≥ Median = 1 | Quartiles | z-score |
| Coffee | Beneficial | Polyphenol-rich beverage associated with favorable microbiota composition | 120 mL/day | ≥ Median = 1 | Quartiles | z-score |
| Chickpeas | Beneficial | Prebiotic legumes promoting beneficial microbial activity | 28 g/day | ≥ Median = 1 | Quartiles | z-score |
| Cranberries | Beneficial | Polyphenol-rich fruit with microbiota-modulating effects | 12 g/day | ≥ Median = 1 | Quartiles | z-score |
| Fermented Dairy | Beneficial | Provides probiotic microorganisms and fermentation products | 105 g/day | ≥ Median = 1 | Quartiles | z-score |
| Dietary Fiber | Beneficial | Enhances microbial diversity | 23 g/day | ≥ Median = 1 | Quartiles | z-score |
| Green Tea | Beneficial | Rich in catechins with prebiotic properties | 220 mL/day | ≥ Median = 1 | Quartiles | z-score |
| Soybeans | Beneficial | Source of isoflavones and fermentable substrates | 30 g/day | ≥ Median = 1 | Quartiles | z-score |
| Whole Grains | Beneficial | beneficial microbial activity food | 62 g/day | ≥ Median = 1 | Quartiles | z-score |
| High-fat Diet | Adverse | Excess dietary fat may impair gut barrier integrity and promote dysbiosis | 34% of total energy | < Median = 1; ≥ Median = 0 | Reverse Quartiles | Reverse z-score |
| Red Meat | Adverse | Associated with microbial metabolites linked to inflammation | 52 g/day | < Median = 1 | Reverse Quartiles | Reverse z-score |
| Processed Meat | Adverse | Associated with unfavorable microbial composition and endotoxemia | 18 g/day | < Median = 1 | Reverse Quartiles | Reverse z-score |
| Refined Grains | Adverse | Low-fiber carbohydrate source associated with reduced microbiota diversity | 125 g/day | < Median = 1 | Reverse Quartiles | Reverse z-score |

Abbreviations: DI-GM, Dietary Index for Gut Microbiota.

*Median values were calculated using the entire study population (n = 800) before case-control comparisons and independent of gestational diabetes status.

Total DI-GM score range: 0–14 points.

**Example of DI-GM Calculation**

| **Component** | **Participant Intake** | **Median Cut-off** | **Score** |
| --- | --- | --- | --- |
| Broccoli | 80 g/day | 65 g/day | 1 |
| Avocado | 0 g/day | 8 g/day | 0 |
| Coffee | 150 mL/day | 120 mL/day | 1 |
| Chickpeas | 40 g/day | 28 g/day | 1 |
| Cranberries | 10 g/day | 12 g/day | 0 |
| Fermented Dairy | 120 g/day | 105 g/day | 1 |
| Dietary Fiber | 28 g/day | 23 g/day | 1 |
| Green Tea | 300 mL/day | 220 mL/day | 1 |
| Soybeans | 35 g/day | 30 g/day | 1 |
| Whole Grains | 90 g/day | 62 g/day | 1 |
| High-fat Diet | 28% energy | 34% energy | 1 |
| Red Meat | 35 g/day | 52 g/day | 1 |
| Processed Meat | 10 g/day | 18 g/day | 1 |
| Refined Grains | 80 g/day | 125 g/day | 1 |
| **Total DI-GM Score** |  |  | **12/14** |

**Interpretation:** This participant achieved a DI-GM score of 12, indicating high adherence to a dietary pattern supportive of gut microbial health. Higher scores represent greater consumption of microbiota-promoting foods and lower consumption of foods potentially associated with gut dysbiosis.
